# Supplementary figures and images for: The effects of compression load to the trunk on lipid metabolism in an inactive phase
Source: PLoS One. 2022 Jul 6;17(7):e0270705. doi: 10.1371/journal.pone.0270705 (PMC9258835; doi:10.1371/journal.pone.0270705)

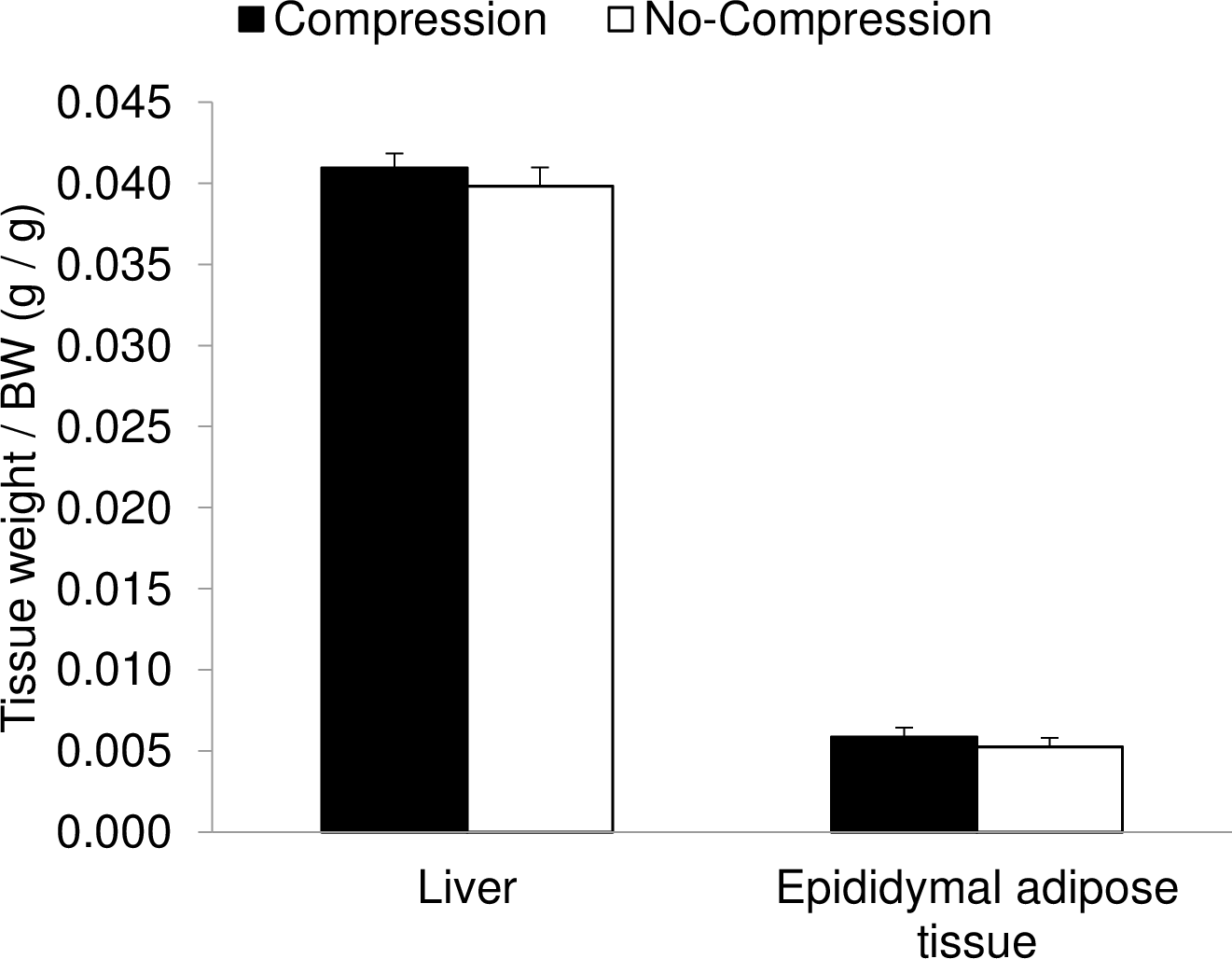

Supplement: S1 Fig — Left, liver: right, epididymal adipose tissue. Values are mean with SEM, n = 7 in each group. (TIF) [file pone.0270705.s002.tif]
